# The evolution of Cow’s Milk-related Symptom Score (CoMiSS™) in presumed healthy infants

**DOI:** 10.1007/s00431-024-05693-2

**Published:** 2024-07-30

**Authors:** Katerina Bajerova, Karolina Hrabcova, Yvan Vandenplas

**Affiliations:** 1 Department of Pediatrics, University Hospital Brno and Faculty of Medicine, Masaryk University, Brno, Czech Republic; 2 Institute of Biostatistics and Analyses, Ltd., Brno, Czech Republic; 3 Vrije Universiteit Brussel (VUB), UZ Brussels, KidZ Health Castle, Laarbeeklaan 101, 1090 Brussels, Belgium

**Keywords:** CoMiSS, Awareness tool, Cow’s milk allergy

## Abstract

The Cow’s Milk-related Symptom Score (CoMiSS™) is a scoring system that reflects the appearance and intensity of symptoms possibly related to consumption of cow’s milk. The original tool was recently updated by changing the cut-off, and the stool scale and by adding angioedema. There is no data available regarding the natural evolution of CoMiSS in infants with no cow’s milk allergy (no-CMA) or a comparison between original and updated CoMiSS values. We determined the original and the updated CoMiSS in infants not diagnosed with cow’s milk allergy. The evolution of CoMiSS during the first year of life was assessed repetitively during predefined check-ups at 1.5, 3, 4, 6, 8, 10, and 12 months. The original and updated scores were compared with the Wilcoxon Signed-Rank Test. We also tested the impact of feeding type, age, gender, and order in the family on the CoMiSS. One hundred and twenty-two infants were included. CoMiSS values during the first year of life showed an inverse relation to age. The difference in CoMiSS between the original and updated versions was significant at 6,8,10, and 12 months (*p* < 0.001), related to the switch from the Bristol Stool Form Scale to the Brussels Infants and Toddlers Stool Scale (BITSS). The difference between both versions of CoMiSS was not significantly different in infants < 6 months (*p* = 0.999 at 1.5 and 4 months, and *p* = 0.586 at 3 months, respectively).

*Conclusion*: CoMiSS decreases with age during the first year of life. While there is no difference between the two CoMiSS versions in healthy infants under 6 months of age, the CoMiSS value in the updated version is lower than the original 1 in infants aged 6 to 12 months.

**Table:** 

What is known? • The Cow’s Milk-related Symptom Score (CoMiSS) is a validated awareness tool for cow’s milk allergy (CMA). • A CoMiSS of ≥ 10 indicates a risk for cow’s milk allergy.
What is new? • The natural evolution of CoMiSS in infants not diagnosed with CMA (no-CMA) shows an inverse relation to age. • There is no difference between the original and the updated CoMiSS versions in no-CMA-infants under six months of age, but the updated CoMiSS is lower in infants 6–12 months than the original one.

What is known?

• The Cow’s Milk-related Symptom Score (CoMiSS) is a validated awareness tool for cow’s milk allergy (CMA).

• A CoMiSS of ≥ 10 indicates a risk for cow’s milk allergy.

What is new?

• The natural evolution of CoMiSS in infants not diagnosed with CMA (no-CMA) shows an inverse relation to age.

• There is no difference between the original and the updated CoMiSS versions in no-CMA-infants under six months of age, but the updated CoMiSS is lower in infants 6–12 months than the original one.

## Introduction

The Cow’s Milk-related Symptom Score (CoMiSS™) is a clinical tool developed to help healthcare professionals (HCPs) be aware that an association of symptoms may indicate the possibility of a cow’s milk allergy (CMA) [1]. Both under- and over-diagnosis of CMA are associated with negative short- and long-term consequences and also have a negative impact on the quality of life [2–5]. It is challenging to diagnose non-IgE mediated CMA, as no laboratory test is available in the clinical setting for this type of allergy. CoMiSS reflects the presence and intensity of five clinical symptoms: crying time, number and volume of regurgitation episodes, stool consistency, skin manifestations (atopic dermatitis/urticaria/angioedema), and respiratory symptoms. The sum of the individual subscores forms the overall result, which varies from 0 to 33 [1]. CoMiSS was updated in 2022 by changing the Bristol Stool Form Scale to the Brussels Infants and Toddlers Stool Scale (BITSS) and adding angioedema to the skin symptoms, with an equal scoring as urticaria (0 or 6) [6]. Furthermore, the cut-off for CoMiSS value signalising the possible risk of CMA was adapted from ≥ 12 to ≥ 10 [6].

Previous studies published in 2018 and 2019 assessed CoMiSS in presumed healthy infants aged up to 6 months, showing a median of 3 [7] in a multi-centric European study and 4 [8] in a Polish cohort, respectively. In 2023, a European multicentre survey in presumed healthy infants aged 6–12 months reported CoMiSS values with a median of 3 [9]. Age at assessment, type of feeding, and country influenced the outcome. CoMiSS is independent of gender [7–9]. All these studies used a single assessment model with a variable number of participants in each age group. Infants with scores above the CoMiSS threshold were not further assessed for CMA. Data on the natural evolution of CoMiSS in a cohort of infants not diagnosed with CMA (no-CMA) are not available.

The Czech paediatric primary care system is based on regular infant follow-up, including clinical check-ups, vaccinations, and, if necessary, medical care in case of acute clinical conditions (infections, adverse reactions, nutritional problems, etc.). The mandatory clinical check-ups are provided at a pre-defined age (1.5, 3, 4, 6, 8, 10, and 12 months) and include a detailed history (diet, symptoms, therapy, type of delivery, etc.), anthropometry, and a complete physical examination. This system allows the collection of detailed information at pre-set time points throughout the first year of life without requiring extra examinations.

The primary endpoint of this study was to assess the natural evolution of CoMiSS during the first year of life in no-CMA infants. The secondary endpoint was to compare the original and the updated versions of CoMiSS.

The Hospital Ethical Committee approved the study. All caregivers participating in the study gave written informed consent to the anonymised use of the infant data.

## Material and methods

This prospective longitudinal observational single-centre study was performed in infants who were repetitively assessed seven times during scheduled (1.5, 3, 4, 6, 8, 10, and 12 months of age) standard clinical check-ups. Exclusion criteria were prematurity, dietary restrictions, known food allergies, chronic disease at enrolment or diagnosed during the study period, and any acute infection at the time of scheduled assessment. Data on gestational age, type of delivery, sex, the order of the child in the family (first/second, third)), age (in days) at each evaluation, type of feeding, and medication and items included in the original and updated CoMiSS were collected. The original and updated CoMiSS values were calculated. Three nurses trained in CoMiSS determined the score. If any uncertainty occurred or data were missing, it was adjusted by the paediatrician assessing the infant and supervising the data collection. No data was missing as this two-step data acquisition model was used at each assessment. All infants underwent a physical evaluation at each visit, including anthropometry (Tables 1, 2 and 3).

**Table 1:** Age and feeding characteristics

Prescheduled assessment group (in months)		1.5	3	4	6	8	10	12
Age at assessment median (days)		44.00	93.00	125.00	185.00	246.00	307.00	368.00
Age at assessment Q1 (days)		42.00	91.00	122.00	183.00	244.00	305.00	366.00
Age at assessment Q3 (days)		45.00	95.00	128.00	188.75	248.00	310.00	370.75
N°	Formula-fed°	5	12	23	0	0	0	0
	Exclusively breastfed°	97	87	81	12	1	0	0
	Breastfed and formula-fed°	20	23	18	0	0	0	1
	Breastfed and solid foods°	0	0	0	80	90	80	75
	Formula-fed and solid foods°	0	0	0	30	31	42	46

Legend: *Q1* quartile 1, *Q3* quartile 3, *No* number of infants receiving each type of feeding; °Type of feeding

**Table 2:** Linear model with mixed effect — the evolution of original and updated CoMiSS

	Fixed effect			Random effect
	Coeficient	95% CI	p-value	SD
Intercept original CoMiSS	7.25	6.99; 7.52	< 0.01	0.80
Updated CoMiSS compared to original CoMiSS	0.32	0.00; 0.64	0.048	-
Change per month of age Original CoMiSS	− 0.5	− 0.53; − 0.46	< 0.001	-
Change per month of age Updated CoMiSS compared to original CoMiSS change	− 0.14	− 0.19; − 0,10	< 0.001	-

Legend: *CI* confidence interval, *SD* standard deviation, Intercept reference value

**Table 3:** Comparison of original and updated CoMiSS values regarding age groups

Prescheduled assessment	Original CoMiSS Median (IQR)	Updated CoMiSS Median(IQR)	p-value*
1.5 months	7.0 (5–8)	7.0 (5–8)	> 0.999
3 months	5.0 (4–7)	5.0 (4–7)	0.586
4 months	5.0 (4–7)	5.0 (4–7)	> 0.999
6 months	4.0 (2–5)	4.0 (0–5)	< 0.001
8 months	2.5 (2–4)	1.0 (0–4)	< 0.001
10 months	2.0 (2–3)	0.0 (0–1)	< 0.001
12 months	2.0 (0–2)	0.0 (0–1)	< 0.001

Legend: *Wilcoxon Signed-Rank Test, *IQR* interquartile ratio

## Statistical analysis

A descriptive statistical analysis was performed. The distribution type of variables was tested using the Shapiro–Wilk test. Descriptive statistics are described using the median (IQR) in non-normally distributed variables. Wilcoxon Signed-Rank Test was used to compare the original CoMiSS values with the updated CoMiSS values. A comparison of CoMiSS values regarding the feeding type at each age group was conducted using the Kruskal–Wallis rank sum test. The linear model with mixed effects was applied to analyse the impact of different variables on CoMiSS values and the evolution of the values. The subject identification number was considered a random effect (because of two measurements in one child — original CoMiSS and updated one). As fixed effects, the variables considered were the binary variable of score type, the continuous variable of month of age, and the interaction of these two variables. In consecutive analyses, variables considered as a fixed effect were feeding type, gender, order in family, and month of predefined assessment (Table 4). The only binary variable was the CoMiSS (original or updated), while the other variables did not have a dichotomous nature but were categorical or continuous. We tested for collinearity among the fixed effects in our mixed model using the variance-inflation factor to address possible collinearity between feeding type and age. The results indicated that collinearity was not an issue in our model.

**Table 4:** Linear model with mixed effect — the effect of different covariates

	Original CoMiSS				Updated CoMiSS			
	Fixed effect			Random effect	Fixed effect			Random effect
	Coeficient	95% CI	*p*-value	SD	Coeficient	95% CI	*p*-value	SD
Intercept	6.20	5.51; 6.89	< 0.001	0.67	6.43	5.68; 7.17	< 0.001	0.62
Formula-fed°	Reference	-	-	-	Reference	-	-	-
Exclusively breastfed°	0.63	0.06; 1.21	0.032	-	0.61	− 0.02; 1.25	0.059	-
Breastfed and solid food°	− 0.78	− 1.42; − 0.14	0.018	-	− 1.52	− 2.23; − 0.80	< 0.001	-
Breastfed and formula-fed°	0.34	− 0.31; 1.00	0.306	-	0.30	− 0.44; 1.03	0.428	-
Formula-fed and solid foods°	− 0.98	− 1.62; − 0.33	0.003	-	− 1.95	− 2.68; − 1.22	< 0.001	-
Girls	Reference	-	-	-	Reference	-	-	-
Boys	− 0.01	− 0.32; 0.30	0.936	-	0.05	− 0.27; 0.38	0.738	-
Order of the infant in the family	0.16	− 0.06; 0.39	0.152	-	0.11	− 0.12; 0.34	0.364	-
Change of CoMiSS per month of age	− 0.33	− 0.39; − 0.27	< 0.001	-	− 0.38	− 0.44; − 0.31	< 0.001	-

Legend: *CI* confidence interval, *SD* standard deviation, Intercept the reference value; °Type of feeding; Order of the infant in the family, firstborn, second or third child in the family

All statistical tests were two-tailed and performed at the *α* = 0.05 significance level. Data were analysed using the R software (computer program; Version 4.1.2; URL https://www.R-project.org/). The sample size calculation was not performed as a similar study was not conducted yet. Moreover, the dropout ratio could not be foreseen.

## Results

**Fig. 1:**
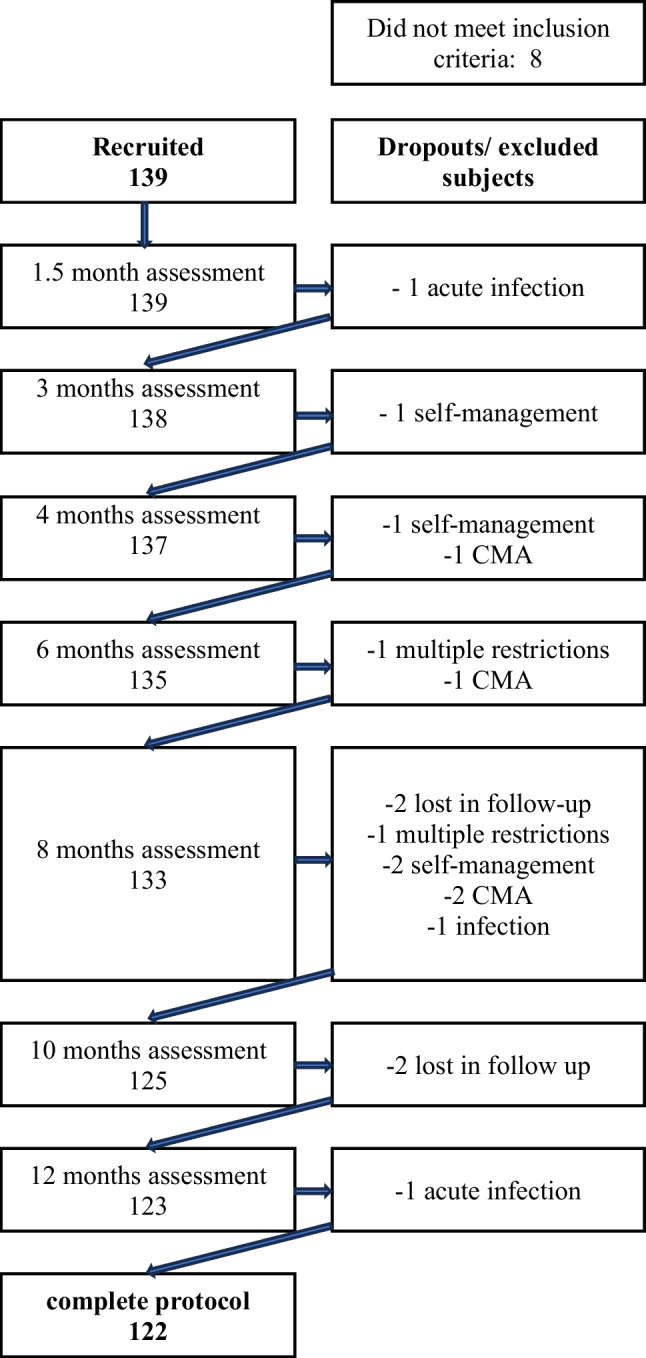
Flow diagram of enrolled and excluded subjects

We recruited 139 infants (74 boys) with a median gestational age of 39 weeks (interquartile range (IQR), 39–40). Forty-one (29.9%) were born by Caesarean section. Seventeen enrolled subjects were then excluded from the analyses for the following reasons: four were diagnosed with CMA by positive challenge (2.9%); another four infants (2.9%) received a cow’s milk-free diagnostic elimination diet due to the symptoms reported by the parents, possibly suggesting CMA (these parents refused the OFC); another two (1.45%) infants were under dietary restrictions started by the parents; in addition three (2.17%) infants presented an acute infection at the time of the scheduled assessment (one with COVID-19, one with Salmonella, and one with pinworms), and finally, four (2.9%) of the initially included infants were lost in follow-up. A total of 122 infants (64 (52.5%)boys) completed the study. These 122 infants were assessed seven times at the pre-defined checkups at the age of 44 (42–45) (median (IQR)) days; 93 (91–95); 125 (122–128); 185 (183–189); 246 (244–248); 307 (305–310), and 368 (366–371) days, respectively. We obtained a total of 854 assessments in 122 infants. The flow diagram is presented (Fig. 1), and the descriptive statistics are summarised in (Table 1).

CoMiSS values in no-CMA infants during the first year of life showed an inverse relation to age: the median (IQR) 7 (5–8) for both the original and updated CoMiSS at 1.5 months decreased to 2 (0–2) for the original and 0 (0–1) for the updated CoMiSS at 12 months (Table 3).

The evolution of both CoMiSS versions when using the linear model is summarised in (Table 2). The intercept is the score value of a child from the reference category at age zero (it is represented by the mean of the original CoMiSS, regardless of other variables) and gains the value of 7.23. The updated CoMiSS is 0.32 points higher than the original one at inclusion. During the first year of life, the original CoMiSS decreases by 0.5 points and the updated by 0.64 points each month (Table 2). Thus, the updated CoMiSS decreases faster. When considering the age groups, the statistically significant but clinically irrelevant difference was found in all age categories in infants older than 6 months (6, 8, 10, and 12 months; *p* < 0.001)), being lower with the updated CoMiSS. In the infants younger than 6 months, there was no difference (assessment at 1.5, 3, and 4 months: *p* > 0.99, *p* = 0.586, and *p* > 0.99) (Table 3). We had no infant presenting with urticaria or angioedema. The type of feeding, when analysed per assessment category, did not influence CoMiSS, except in the 6-month assessment group, where a difference between feeding types was found with the original (*p* = 0.023; Kruskal–Wallis rank sum test) as well as the updated CoMiSS (*p* = 0.003), related to the introduction of solids. The original CoMiSS shows that an exclusively breastfed infant has a 0.63-point higher score than a formula-fed infant. After the introduction of solid foods in exclusively breastfed infants, the CoMiSS decreased by 0.78 in comparison to the previous value. The introduction of solid foods in formula-fed infants led to a decrease of 0.98. Similar results were obtained for the updated CoMiSS: solid food introduction in a breastfed baby reduces CoMiSS by 1.5 and in a formula-fed infant by 1.95. The original CoMiSS decreased by 0.33 points, and the updated CoMiSS by 0.38 points with each month age increased. Gender and order of the infant in the family did not influence CoMiSS (Table 4).

## Discussion

CoMiSS was determined repetitively in 122 no-CMA infants during the first year of life and showed an inverse relation to age: median 7 for both the original and updated CoMiSS at 1.5 months, 2 original at 12 months, and 0 updated CoMiSS, respectively. Original and updated CoMiSS do not differ in infants younger than 6 months (*p* = 0.586 at 3 months and 0.999 at 1.5 and 4 months). In comparison to data obtained from previously presumed healthy infants aged up to 6 months [7, 8] (Table 5), our observed median values are higher due to loose and watery stools, which were predominant until the introduction of solids, leading to increased stool consistency and decreased CoMiSS. This finding is similar to Bigorajska's observation[8]. This is the first study using a longitudinal repeated assessment of CoMiSS in no-CMA infants. Previous studies in presumed healthy infants applied a single assessment mode [7–9]. Each age group included different numbers of infants (Table 5). Only one study (focused initially on interrater and day-to-day variability) reported CM challenge in presumed healthy infants with CoMiSS values ≥ 10 [10].

**Table 5:** Age-appropriate values — comparison with previous studies

	Age	No	Min	P05	P25	Median	P75	P95	Max
Bigorajska [8]	1 mo	28	3	3	5	6.5	9	13.3	15
Vandenplas [7]	1–2 mo	129	0	0	2	4	6	10	14
Bajerova oC	1.5 mo	122	4	4	5	7	8	10	12
Bajerova uC	1.5 mo	122	4	4	5	7	8	10	12
Vandenplas	2–3 mo	94	0	0	2	4	6	9	10
Bigorajska	3 mo	55	0	0	1	3	5	9.3	14
Bajerova oC	3 mo	122	2	4	4	5	7	9	13
Bajerova uC	4 mo	122	1	4	4	5	7	9	13
Vandenplas	3–4 mo	88	0	0	1	4	6	11	15
Bigorajska	4 mo	72	0	0	2	4	7	11.5	15
Bajerova oC	4 mo	122	2	4	4	5	7	8.9	10
Bajerova uC	4 mo	122	0	4	4	5	7	8.9	10
Vandenplas	4–6 mo	113	0	0	1	3	5	8	12
Bigorajska	6 mo	15	0	0	0.5	4	4.5	9	9
Bajerova oC	6 mo	122	0	0	2	4	5	6	11
Bajerova uC	6 mo	122	0	0	0	4	5	6	11
Jankiewicz [9]	6 mo	137	0	0	1	4	6	9	12
Jankiewicz	7mo	105	0	0	1	3	5	8	10
Jankiewicz	8mo	104	0	0	1	3.5	5	6.85	10
Bajerova uC	8 mo	122	0	0	0	1	4	5.9	8
Bajerova oC	8 mo	122	0	0	2	2.5	4	5.9	6
Jankiewicz	9 mo	84	0	0	1	3	5	8.7	12
Jankiewicz	10 mo	78	0	0	0.25	2	4	6	8
Bajerova uC	10 mo	122	0	0	0	0	1	4	7
Bajerova oC	10 mo	122	0	0	2	2	3	4	9
Jankiewicz	11 mo	80	0	0	0	2	4	7	9
Jankiewicz	12 mo	21	0	0	0	1	4	7	9
Bajerova uC	12 mo	122	0	0	0	0	1	4	5
Bajerova oC	12 mo	122	0	0	0	2	2	4	5

Legend: *oC* original CoMiSS, *uC* updated CoMiSS

The decrease in CoMiSS was more pronounced with the updated CoMiSS (− 0.64 per month) than with the original CoMiSS (dropped by 0.5 per month). Age and type of feeding, such as the introduction of solids, impact the evolution of CoMiSS in this cohort.

The effect of age on CoMiSS values in presumed healthy infants was analysed in three studies [7–9]. The European study (data from four countries, infants up to 6 months) detected a trend towards differences across ages, with higher scores in the 1–2 months and 3–4 months age groups (*p* < 0.01). Also, in the study carried out in Poland with identical enrolment parameters [8], age had an impact on the total CoMiSS (*p* < 0.001). However, the number of infants included in some age groups was small (15 subjects), complicating the comparison of outcomes. A study in 609 European infants aged 6 to 12 months showed higher total CoMiSS in infants aged 6 months compared to 10 months (*p* = 0.001). Furthermore, the highest median CoMiSS was observed in 6-month-old infants, while the lowest median value was observed at 12 months [9]. These studies used a single assessment model — the number of included subjects and assessments was identical. The comparison of age-appropriate CoMiSS values, including our data, is summarised in Table 5.

The type of feeding did not impact the CoMiSS in the European cohort study (*p* = 0.43) [7]. However, the type of feeding (exclusive breastfeeding, formula, mixed feeding) influenced CoMiSS (*p* < 0.001) in the Polish study [8]: the median (Q1–Q3) CoMiSS showed a trend to be higher, with more prevalent loose stools in breastfed than in formula-fed infants (median (IQR) 4.0 (2–7) vs 3.0 (1–4), respectively). No difference was found regarding the type of feeding in Polish infants aged 6–12 months [9].

Original and updated CoMiSS observed in this study does not differ significantly in infants under 6 months of age, although there was a decreasing trend with a median (IQR) of 7.0 (5–8) at 1.5 months dropping to 5 (4–7) at 6 months. This finding aligns with the study comparing the CoMiSS, including the Bristol Stool Form Scale (BSFS) and the CoMiSS values obtained by substituting the stool scale with the Brussels Infants and Toddlers Stool Scale (BITSS) [11], reporting a difference in CoMiSS-BSFS and CoMiSS-BITSS for values ≤ 5 (*p* < 0.001), but values ≥ 6 remained unchanged (*p* = 0.81) [11].

This is a single-centre study in Czech infants. Our outcomes may differ from those of other countries, as there might be a difference between CoMiSS in different countries [7]. We cannot rule out that the natural evolution of CoMiSS values may also differ in relation to the region.

The limitation of this study is that it is a single-centre observation and has an uneven distribution of feeding types, as there is a low ratio of non-breastfed infants (4.1%). In 2021, 3452 out of 103.068 with birthweight ≥ 2500 g were not breastfed (3.4%) [12]. The eight infants suspected to have developed CMA were treated with a CM-free diagnostic elimination. Unfortunately, four parents declined the CM oral food challenge because “that would make the child sick again”. The infants who were put on dietary restrictions by the parents were excluded because we could not confirm that these infants were no-CMA.

## Conclusion

This study demonstrates the evolution of CoMiSS in no-CMA infants during the first year of life. The overall trend of CoMiSS during the first year of life showed an inverse relation to age, which is more pronounced with updated CoMiSS. There was no difference in CoMiSS in infants under 6 months of age. The original and updated CoMiSS in our cohort differed after 6 months of age, related to the difference in stool consistency.

## Data Availability

No datasets were generated or analysed during the current study.

## Abbreviations

BITSS: Brussels Infants and Toddlers Stool Scale
BSFS: Bristol Stool Form Scale
CMA: Cow’s milk allergy
No-CMA: Not diagnosed with CMA
CoMiSS: Cow’s Milk-related Symptom Score
HCP: Health care professional
IQR: Interquartile ratio
